# Dusp16 Deficiency Causes Congenital Obstructive Hydrocephalus and Brain Overgrowth by Expansion of the Neural Progenitor Pool

**DOI:** 10.3389/fnmol.2017.00372

**Published:** 2017-11-09

**Authors:** Ksenija Zega, Vukasin M. Jovanovic, Zagorka Vitic, Magdalena Niedzielska, Laura Knaapi, Marin M. Jukic, Juha Partanen, Roland H. Friedel, Roland Lang, Claude Brodski

**Affiliations:** ^1^Department of Physiology and Cell Biology, Zlotowski Center for Neuroscience, Faculty of Health Sciences, Ben-Gurion University of the Negev, Beersheba, Israel; ^2^Institute of Clinical Microbiology, Immunology and Hygiene, University Hospital Erlangen, Friedrich-Alexander-Universität Erlangen-Nürnberg, Erlangen, Germany; ^3^Department of Biosciences, University of Helsinki, Helsinki, Finland; ^4^Departments of Neuroscience and Neurosurgery, Friedman Brain Institute, Icahn School of Medicine at Mount Sinai, New York, NY, United States

**Keywords:** DUSP16, hydrocephalus, brain overgrowth, megalencephaly, macrocephaly, neurogenesis, neural differentiation, neuronal progenitors

## Abstract

Hydrocephalus can occur in children alone or in combination with other neurodevelopmental disorders that are often associated with brain overgrowth. Despite the severity of these disorders, the molecular and cellular mechanisms underlying these pathologies and their comorbidity are poorly understood. Here, we studied the consequences of genetically inactivating in mice dual-specificity phosphatase 16 (*Dusp16*), which is known to negatively regulate mitogen-activated protein kinases (MAPKs) and which has never previously been implicated in brain development and disorders. Mouse mutants lacking a functional *Dusp16* gene (*Dusp16*^−/−^) developed fully-penetrant congenital obstructive hydrocephalus together with brain overgrowth. The midbrain aqueduct in *Dusp16*^−/−^ mutants was obstructed during mid-gestation by an expansion of neural progenitors, and during later gestational stages by neurons resulting in a blockage of cerebrospinal fluid (CSF) outflow. In contrast, the roof plate and ependymal cells developed normally. We identified a delayed cell cycle exit of neural progenitors in *Dusp16*^−/−^ mutants as a cause of progenitor overproliferation during mid-gestation. At later gestational stages, this expanded neural progenitor pool generated an increased number of neurons associated with enlarged brain volume. Taken together, we found that *Dusp16* plays a critical role in neurogenesis by balancing neural progenitor cell proliferation and neural differentiation. Moreover our results suggest that a lack of functional *Dusp16* could play a central role in the molecular mechanisms linking brain overgrowth and hydrocephalus.

## Introduction

Hydrocephalus, estimated to affect 1 in 1000 infants is a condition caused by abnormal cerebrospinal fluid (CSF) flow that leads to progressive ventricular dilatation and neural damage. Most infantile hydrocephalus cases are congenital (Tully and Dobyns, 2014). Based on the CSF dynamics, hydrocephalus can be classified as obstructive, resulting from impeded CSF circulation or non-obstructive, caused by increased CSF production or impaired CSF resorption (McAllister, 2012; Tully and Dobyns, 2014; Kahle et al., 2016; Kousi and Katsanis, 2016). Congenital hydrocephalus is particularly difficult to treat and often result in the poorest neurological outcomes (Kahle et al., 2016). Although obstruction of CSF flow is the most common culprit in congenital hydrocephalus, the molecular and cellular underpinnings of the occlusion are not well understood (McAllister, 2012; Tully and Dobyns, 2014).

Brain overgrowth has been implicated in a broad range of neurodevelopmental disorders. Pathohistological changes in such conditions can be either prominent, as in megalencephaly syndromes (i.e., brain weight ≥2 standard deviations above mean), or, subtle, as in autism spectrum disorders (Mirzaa and Poduri, 2014; Hevner, 2015; Winden et al., 2015). Mutations affecting growth factor signaling pathways including mTOR and RAS/mitogen-activated protein kinase (MAPK) pathways have been identified as major causes of brain overgrowth disorders (Hevner, 2015). Interestingly, there is a known co-morbidity of brain overgrowth disorders with hydrocephalus including megalencephaly syndromes, RASopathies and autism spectrum disorders (Fernell et al., 1991; Lindquist et al., 2006; Mirzaa and Poduri, 2014; Winden et al., 2015; Kousi and Katsanis, 2016). However, little is known about the genetic and cellular mechanisms combining these two pathologies.

Neural progenitor proliferation during embryonic neurogenesis needs to be tightly regulated, since it is fundamental for proper brain function. Consequently, dysregulation in the numbers of forming neurons can cause a broad spectrum of brain disorders. Neural progenitors initially divide symmetrically to expand their pool and switch to neurogenic division at the onset of neurogenesis (Götz and Huttner, 2005). The balance between self-renewal and cell cycle exit of neural progenitors, and the generation of the appropriate numbers of postmitotic progeny is critical for normal brain formation. This process is regulated by a set of growth factors triggering different signaling cascades, including the NOTCH, WNT, SHH, BMP and Fibroblast growth factor (FGF) pathways (Götz and Huttner, 2005; Paridaen and Huttner, 2014). These cascades maintain extensive cross talks, whereby MAPKs plays a prominent role in integrating different signaling pathways (Miloso et al., 2008).

Dual-specificity phosphatases (DUSPs) are a group of proteins that can dephosphorylate both phosphotyrosine and phosphoserine/phosphothreonine residues within one substrate and are major modulators of central signaling pathways. The best characterized group of DUSPs are MAPK phosphatases, which currently include 10 members. MAPK phosphatases that are activated by different extracellular signals, including growth factors, play a critical role in MAPK pathways by negatively regulating ERK, JNK and p38 MAPK activity via de-phosphorylation (Dickinson and Keyse, 2006; Patterson et al., 2009).

Functions of DUSPs in cancer and immune response are well documented (Dickinson and Keyse, 2006; Patterson et al., 2009). However, few DUSPs have been associated with neural development. DUSP1 and -6 are implicated in cell survival, cell death and axonal development (Jeanneteau et al., 2010; Finelli et al., 2013; Collins et al., 2015). Recently DUSP16 was identified as an axonal preserving factor during development (Maor-Nof et al., 2016). In contrast, for most other DUSPs there are no data available on their role in the formation of the nervous system (Bermudez et al., 2010). Interestingly, in each of these reports, DUSP activity was mediated by modifying the activity of ERKs rather than JNK or p38 MAPK.

DUSP16 preferentially dephosphorylates JNKs and p38 MAPK (Masuda et al., 2001; Matsuguchi et al., 2001), and has been implicated in carcinogenesis and in maintaining a balanced immune response by modulating the magnitude and duration of effector functions of innate and adaptive immune cells (Niedzielska et al., 2014). *Dusp16*-deficient gene trap mice die perinatally without gross developmental abnormalities (Niedzielska et al., 2014). However, the role of *Dusp16* in CNS morphogenesis or disorders was not previously explored.

Here we report that the genetic inactivation of *Dusp16* leads to congenital obstructive hydrocephalus and brain overgrowth. The neural progenitor pool was increased In *Dusp16*^−/−^ mutants, due to a delay in neural differentiation. The expanded neural progenitors obstructed the midbrain aqueduct, and later during embryogenesis give rise to an increased number of neurons causing brain overgrowth.

## Materials and Methods

### Breeding of Mouse Mutant Colony

All mice were housed in a temperature-controlled (21–23°C) environment, under a 12-h light/dark cycle and had free access to food and water in a pathogen-free animal facility. All procedures and experimental protocols conducted on the animals were approved by the Institutional Animal Care and Ethics Committee at Ben-Gurion University of the Negev (Approval # IL-53-09-2016). The generation of *Dusp16* gene trap mouse line has been described in detail elsewhere (Niedzielska et al., 2014). *Dusp16* gene trap mouse mutants (*Dusp16*^−/−^) were kept in a pure C57BL/6 genetic background and die perinatally as homozygotes (Niedzielska et al., 2014). C57BL/6 mice heterozygous for *Dusp16* (*Dusp16*^+/−^) were mated to obtain homozygous-deficient *Dusp16* embryos.

Genotyping of adult animals and embryos for the *Dusp16* locus and the *β-Geo* expression cassette was performed by polymerase chain reaction (PCR) analysis on gDNA isolated from tail biopsies as described previously (Niedzielska et al., 2014). The sequences of the primers for *Dusp16* genotyping are: RL528: 5′-CGCTCTTACCAAAGGGCAAACCC-3′, RL535: 5′-CCATCTCATGGCAGAGGAGTGACT-3′, RL536: 5′-GACCTGTGCATAACTGGCCCTACTAC-3′. The expected size for the PCR product of a wild type (WT) allele is 450 bp, the size of the *LacZ* knock-in allele is 650 bp.

### BrdU Administration

To label cells during the S-phase of the cell cycle, the DNA synthesis marker, 5-bromo-2′-deoxyuridine (BrdU; Sigma, cat # B5002) was dissolved in sterile 0.9% NaCl solution at a concentration of 10 mg/ml. Timed mated pregnant females were given a single intraperitoneal (i.p.) injection of BrdU solution at a concentration of 50 mg of BrdU/kg of body weight. Pregnant females were sacrificed and embryos were collected 1 h after the i.p. injection (to label cells during S-phase) or 24 h after the i.p. injection (to label cells in S-phase and postmitotic cells that exited the cell cycle in the previous 24 h) and processed for paraffin embedding according to standard protocols.

### Dissection, Embedding and Histology of Mouse Embryos

Heterozygous females were caged together with heterozygous males during the 12-h dark period. The day of vaginal plug detection was considered as day 0.5. Timed mated pregnant females at different stages of pregnancy from E11.5 to E18.5 were euthanized by isoflurane. Embryos were dissected free of maternal tissues in cold phosphate-buffered saline pH 7.4 (1× PBS), decapitated and heads (E11.5–E15.5) or brains (E16.5–E18.5) were placed in individual tubes and fixed in 4% paraformaldehyde in 1× PBS overnight at 4°C. Following fixation, tissue samples were washed in 1× PBS, dehydrated through the series of graded ethanol, isopropanol and toluene to displace the water, and then infiltrated with Paraplast Plus tissue embedding medium (Leica). For the embryonic phenotype analysis only homozygous *Dusp16*^−/−^ were used. WT littermates were used as a control for *Dusp16*^−/−^ mutants.

For histological analysis, paraffin blocks were sectioned at 5 μm in thickness and stained with Hematoxylin/Eosin (H&E) by standard methods. Tissue morphology was evaluated by light microscopy (Olympus U-PMTVC, Japan). Representative sections are shown based on at least three animals per genotype group.

### LacZ Staining Using X-Gal

Heterozygous *Dusp16*^+/*−*^ and WT littermate embryos were generated through mating of heterozygous *Dusp16*^+/−^ male with WT females. Pregnant females at different stages of pregnancy were sacrificed and E11.5 or E12.5 embryos were dissected in 1× PBS. Embryos were decapitated and fixed in 4% PFA/PBS for 1 h. Heads of embryos were then washed in 1× PBS followed by 3 × 20 min washes in detergent wash solution (2 mM MgCl2, 0.01% sodium deoxycholate, 0.02% NP40 in 1× PBS pH 7.4). Detergent wash solution was replaced by X-gal staining solution (1 mg/ml X-gal, 5 mM potassium ferricyanide, 5 mM potassium ferrocyanide in detergent wash solution) overnight at 37°C.

The following day, the staining solution was removed and samples were washed in 1× PBS, post-fixed in 4% PFA/PBS for 3 h and again washed in 1× PBS. Fixed embryos were then imaged with a microscope or embedded in 4% low melting agarose (Sigma Aldrich) and processed for vibratome sectioning (100 μm thickness). Slices were mounted on SuperfrostPlus® glass slides (Thermo Scientific), glued with Immu-Mount™ (Thermo Scientific) and evaluated using Olympus U-PMTVC microscope.

### Immunohistochemistry on Paraffin Sections

For immunohistochemistry analysis, paraffin sections (5 μm) were first de-waxed using xylene washes (3 × 5 min each), subsequently re-hydrated in 100% ethanol (2 × 5 min each), followed by decreasing series of ethanol (95%, 70%, 50%, 1 × 5 min each) and ddH2O (2 × 5 min each). After antigen retrieval in boiling citric acid (pH 6) for 10 min, sections were blocked in 4% normal goat serum (NGS) for 1 h and incubated in primary antibodies diluted in blocking buffer overnight at 4°C. Primary antibodies used: mouse anti BrdU (1:500, Sigma B2531), mouse anti BRN-3A (1:300, SantaCruz sc-8429), rabbit anti CAS-3 (1:200, Cell Signaling D175), rabbit anti CCND2 (1:200, SantaCruz sc-593), rabbit anti KI67 (1:100, Abcam ab16667), rabbit anti LMX1A (1:400, Millipore AB10533), rabbit anti LMX1b (1:1000, gift from Dr. C. Birchmeir, Germany), rabbit anti MAP-2 (1:100, SantaCruz sc20172), mouse anti N-CAD (1:200, BD 610920), mouse anti Nestin (1:10, DSHB rat-401), mouse anti NEUN (1:100, CHEMICON MAB377x), rabbit anti OCLN (1:25, Invitrogen 71-1500), mouse anti p-ERK (1:100, Sigma M9692), rabbit anti p-JNK (1:100, Promega V793B), rabbit anti p-P38 (1:1000, Cell Signaling 4511), rabbit anti Reissner’s fiber (RF; 1:200, kindly provided by Dr. Esteban Rodriguez, Austral University of Chile), rabbit anti RFX3 (1:300, Sigma HPA035689), rabbit anti Zonula occludens-1 (ZO-1; 1:100, Invitrogen 40-2200), rabbit anti PHH3 (1:1000, Millipore 06-570), rabbit anti-SOX2 (1:400 Millipore AB5603), mouse anti-HUC/D (1:800 Molecular probes A-21271). The next day, sections were washed for in 1× TBST and incubated in Fluorophore conjugated (Cy2- and/or Cy3-, 1:100, Jackson Laboratories) secondary antibodies diluted in 1× TBST, for 1 h at room temperature in the dark. Finally, sections were washed in 1× TBST and mounted using regular Immu-Mount™. Biotinylated secondary antibodies were visualized with the Vectastain ABC immunoperoxidase kit (Vector Laboratories, PK-6200) using 3,3′-diaminobenzidine tetrahydrochloride (DAB) substrate.

For detection of BrdU incorporation, sections were re-hydrated and antigen retrieved as described above. After antigen retrieval step, sections were immediately treated with 2N HCl for 30 min at 37°C. Sections were then washed in 1× PBS/0.05% Tween-20, incubated in 1× PBS/0.5% Triton for 1 h and blocked in blocking buffer (1% BSA in 1× PBS/0.05% Tween-20) for 1 h at room temperature, and incubated in anti-BrdU antibody diluted in blocking buffer overnight at 4°C.

### mRNA *in Situ* Hybridization

For mRNA *in situ* probes for *Dusp16*, the sequence that had been published by GenePaint^1^ was used. *Dusp16* sequence of 1012 bp was amplified from cDNA pool by PCR using following primers: Forward primer: 5′-CACTCAGATATTCTGGCTCCC-3′, Reverse primer: 5′-CTAGACATGGTAGTGGTGATGGC-3′. Cloning of the PCR product into the plasmid was performed using pGEM®-T Easy Vector (Promega, A1360) following the manufacturer’s standard protocol. Standard *in vitro* transcription reactions for *in vitro* RNA synthesis (sense and antisense) were performed using 10× DIG RNA Labeling Mix following the manufacturer’s standard protocol. Tissue preparation and *in situ* hybridization were processed as described previously (Stylianopoulou et al., 2012; Sherf et al., 2015).

### Total Brain Tissue Surface Quantification

For measurement of total brain tissue surface, H&E serial coronal sections (5 μm) throughout the entire brain (every 50 μm of E12.5 and E13.5 and every 60 μm of E16.5) were captured with an Olympus U-PMTVC microscope (Japan). Total surface area was quantified using ImageJ software by outlining the entire brain surface, excluding the ventricles, and then applying the area measurement function.

### Thickness of the Cortex Quantification

The thickness of the cortical layers was measured at the level of the dorsal hippocampus (future sensorimotor cortex) as described previously (Bible et al., 2004). Using a 5× objective, three consecutive H&E coronal sections (5 μm) were captured. Cortical region boundaries were defined by straight lines extending perpendicularly from the corticostriatal boundary and the dorsomedial apex to the pial surface. Ten lines placed on each of three consecutive sections spanning each cortical region were made. The length of perpendicular lines extending from the ventricular surface to the pial surface was measured using Adobe Illustrator software.

### Cell Counting

For E12.5 embryos, the total number of cells (BrdU) was obtained by counting cells from every tenth section at 5 μm thickness. For E13.5 embryos, cells from one-half of the sections separated at the dorsal midline were counted on every tenth section (BRN3A) or every twentieth section (quitting fraction) at 5 μm thickness. For quantification of phosphorylated p38^+^ MAPK cells in the region of occlusion demarked by the posterior commissure (PC) at E13.5, all cells present in ventricular zone (VZ) were counted on every tenth section at 5 μm thickness. For E18.5 embryos, the total number of cells (BRN3A) from one-half of the sections separated at the dorsal midline was counted on every 56th (BRN3A) at 5 μm thickness. The total number of positively labeled cells was determined by summing the values in all tissue sections.

### Statistical Analysis

Two-tailed paired sample Student’s *t*-test and two-tailed unpaired Student’s *t*-test were performed to test the significance of difference in numerical data as appropriate. The data in the text and the error bars on the figures represents mean ± standard error of the mean (SEM). Every significant change and interaction was marked by an asterisk between the bars (*p < 0.05, **p < 0.01 and ***p < 0.001).

## Results

### Dusp16 Is Highly Expressed in the Ventricular Zone of the Embryonic Mouse Brain, Suggesting its Implication in Neurogenesis

In order to study the role of *Dusp16* in neural development, we visualized its expression pattern during embryogenesis. To do so, we used mouse mutants with a beta-Galactosidase-Neomycin transferase (beta-Geo) gene trap insertion in the *Dusp16* locus, generating a *Dusp16*-beta-Geo mRNA (Niedzielska et al., 2014). The enzymatic activity of the protein encoded by this transcript was used to visualize the pattern of *Dusp16* expression in E11.5 and E12.5 and E15.5 embryos heterozygous for *Dusp16* (*Dusp16*^+/−^). Adult *Dusp16*^+/−^ mice are viable and fertile without apparent morphological or behavioral changes (Niedzielska et al., 2014). We combined the analysis of these heterozygote mutants with mRNA *in situ* hybridization on WT animals performed by us and a publicly available database (Magdaleno et al., 2006).

**Figure 1:**
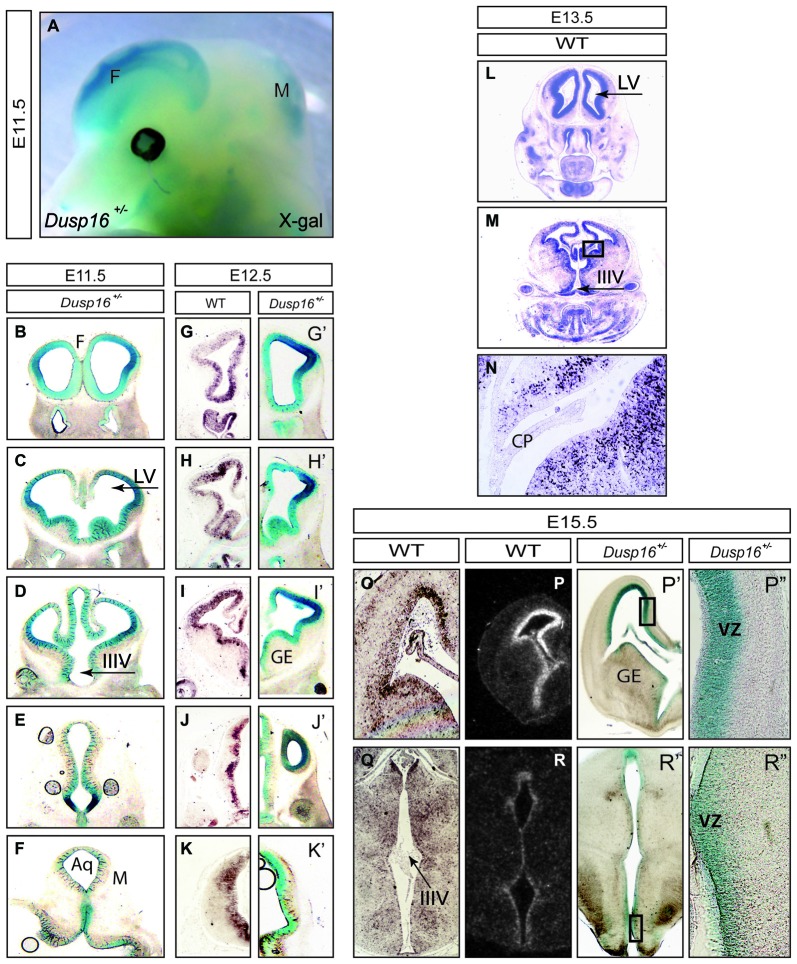
Dual-specificity phosphatase 16 (*Dusp16*) is highly expressed in the VZ of the embryonic mouse brain. **(A)** X-Gal staining of an E11.5 embryo head expressing the *LacZ* gene under the endogenous *Dusp16* promoter. *Dusp16* is strongly expressed in the forebrain and dorsal midbrain at E11.5. **(B–K′)** Serial rostral to caudal coronal sections of E11.5 and E12.5 *Dusp16*^+/−^ embryos demonstrate strong expression of *Dusp16* in the VZ of developing brains shown by lacZ staining and mRNA *in situ* hybridization. **(L–N)** mRNA *in situ* hybridization using a *Dusp16* mRNA probe demonstrates that *Dusp16* continues to be expressed also at E13.5 and E15.5 **(O–R)**, that corresponds to lacZ staining at E15.5 **(P′,P″,R′,R″)**. CP, choroid plexus; F, forebrain; IIIV, third ventricle; LV, lateral ventricle; M, midbrain; VZ, ventricular zone; GE, ganglionic eminence; Aq, Aqueduct of Sylvius.

At E11.5 expression of β-galactosidase was abundant in the CNS, particularly in the forebrain and dorsal midbrain (Figure 1A). Serial rostral (R) to caudal (C) coronal vibratome sections of X-Gal stained *Dusp16*^+/*−*^ mouse heads showed that β-galactosidase was expressed in the VZ of the developing brain (Figures 1B–K). The absence of endogenous β-galactosidase activity was demonstrated in WT littermates that underwent the same protocol in parallel but failed to show any signal (data not shown). At E12.5 we complemented X-Gal stainings (Figures 1G′–K′) with mRNA *in situ* hybridization (Figures 1G–K) performed in our lab and found that both methods demonstrated a consistent expression pattern of *Dusp16* in the VZ of the developing brain at this stage. At E13.5 *Dusp16* mRNA was expressed predominantly in the VZ of the developing brain (Figures 1L,M) but not in the choroid plexus (CP; Figure 1N). Also at E15.5, *Dusp16* gene expression was mostly confined to the VZ as seen in our mRNA *in situ* hybridization experiments (Figures 1O,Q), the publicly available database (Figures 1P,R) and our X-Gal stainings (Figures 1P′,P″,R′,R″). In summary, *Dusp16* was highly expressed in the VZ of the forming brain, suggesting a potential role for this gene in modulation of neurogenesis.

### Aqueductal Stenosis Causes Congenital Obstructive Hydrocephalus in *Dusp16*^−/−^ Mutants

**Figure 2:**
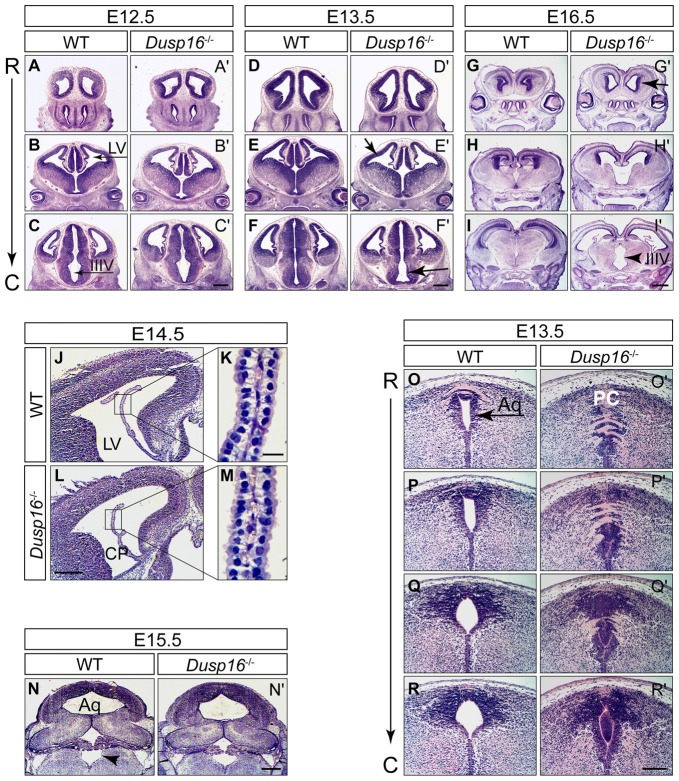
Aqueductal stenosis causes congenital obstructive hydrocephalus in *Dusp16*^−/−^ mutants. Hematoxylin/Eosin (H&E) serial rostral (R) to caudal (C) coronal sections from E12.5, E13.5 and E16.5 wild types (WTs) and *Dusp16*^−/−^ mutants are shown with each pair of sections representing approximately the same coronal plane. **(A–C′)** At E12.5, *Dusp16*^−/−^ mutants show no changes in the ventricular size. **(D–F′)** At E13.5 a subtle enlargement of the lateral and third ventricle starts to be visible in *Dusp16*^−/−^ mutants. **(G–I′)** At E16.5 enlargement of the ventricles are clearly visible (arrows). Scale bars: **(C′,F′)** 500 μm; **(I′)** 200 μm. **(J–M)** H&E staining of coronal hemisections through LV of WT and *Dusp16*^−/−^ brains at E14.5. The overall morphology of CP appears normal in *Dusp16*^−/−^ mutants **(L,M)** compared with control littermates **(J,K)**. Higher magnification of the areas marked by boxes shows that cytoplasmic volume of the CP epithelial cells in *Dusp16*^−/−^ mutants** (M)** is unperturbed compared with WT **(K)**. **(N,N′)** H&E staining of coronal sections through the fourth ventricle of WT and *Dusp16*^−/−^ heads at E15.5 demonstrating the same size of the fourth ventricle in *Dusp16*^−/−^ compared with WT (arrowhead). **(O–R′)** H&E staining of coronal sections through the Aq of WT and *Dusp16*^−/−^ heads at E13.5 revealed a near complete obstruction in *Dusp16*^−/−^ mutants. Scale bars: **(L)** 500 μm; **(K)** 20 μm; **(N′)** 500 μm; **(R′)** 200 μm. Aq, Aqueduct of Sylvius; C, caudal; LV, lateral ventricle; IIIV, third ventricle; PC, posterior commissure; R, rostral; CP, choroid plexus.

In order to investigate the functional relevance of the *Dusp16* expression *in vivo*, we studied brain development in mice lacking a functional *Dusp16 gene* (*Dusp 16*^−/−^). Homozygous *Dusp16*^−/−^ mutants develop without gross morphological abnormalities in organ systems outside the CNS, and die shortly after birth of unknown causes (Niedzielska et al., 2014). Brain ventricles of *Dusp16*^−/−^ mutants at E12.5 exhibited no obvious morphological abnormalities (Figures 2A–C′). However, starting from E13.5, mutants showed enlarged lateral and third ventricles (Figures 2D–F′). At E16.5 massive dilatation of the lateral as well as third ventricle was apparent in *Dusp16*^−/−^ mutants (Figures 2G–I′). The phenotype was fully penetrant.

Congenital hydrocephalus can be caused by increased production of CSF by the CP, impaired absorption of CSF in the blood stream, or as in the majority of congenital cases, by obstruction of CSF flow (McAllister, 2012; Tully and Dobyns, 2014; Kahle et al., 2016; Kousi and Katsanis, 2016). To study whether *Dusp16* could be involved in the development and function of the CP, we analyzed its expression there at E13.5 mRNA *in situ* hybridization did not reveal *Dusp16* expression in the CP of WT embryos (Figure 1N). Moreover, the overall morphology of the CP at E14.5 was normal in *Dusp16*^−/−^ mutants (Figures 2J–M), thereby suggesting that overproduction of CSF is unlikely to have caused the observed hydrocephalus. In *Dusp16*^−/−^ mutants at E15.5 dilatation of the fourth ventricle was not seen (Figures 2N,N′), though enlargement of the lateral and third ventricles were already visible at this developmental stage. This suggests that impaired reabsorption of CSF into the venous blood stream was not responsible for the hydrocephalus in these animals. In contrast, the midbrain aqueduct of Sylvius (Aq) was obstructed from 13.5 onwards compared to the open aqueduct in WTs (Figures 2O–R′). In conclusion, these findings indicate that the hydrocephalus observed in the *Dusp16*-deficient embryos was caused by aqueductal stenosis.

### *Dusp16*^−/−^ Mutants Show an Increase in Brain Parenchyma

**Figure 3:**
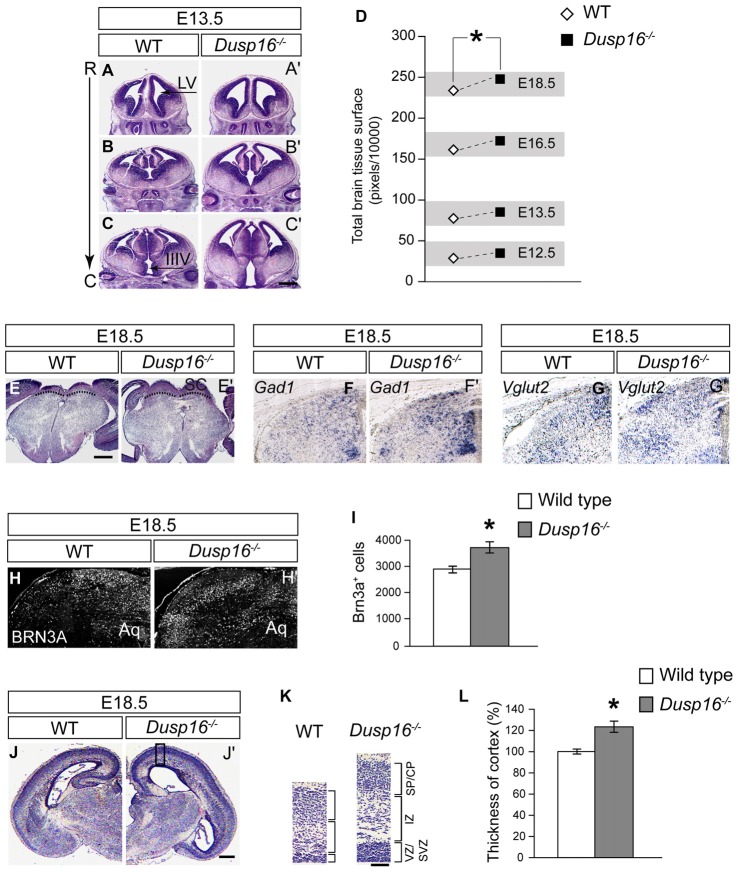
*Dusp16*^−/−^ mutants show an increase in brain tissue.** (A–C′)** Representative H&E staining of WT and *Dusp16*^−/−^ head sections along the rostro-caudal axis (R->C) at E13.5 indicates an increase in neural tissue in *Dusp16*^−/−^ mutants. **(D)** A two tailed paired-samples *t*-test shows a significant increase in the total brain surface, excluding the ventricles, of *Dusp16*^−/−^ mutants at different embryonic stages compared with control littermates. **(E,E′)** H&E coronal sections of the mesencephalon illustrate enlarged superior colliculus of E18.5 *Dusp16*^−/−^ mutants compared with WT (doted lines). **(F,F′)** Hemisections visualizing gabaergic neurons using *Gad1* mRNA probes for *in situ* hybridization indicate that there is an increase in *Gad1* signal strength in mutants. **(G,G′)** Hemisections visualizing glutamatergic neurons using *Vglut2* mRNA probes for *in situ* hybridization indicate that there is an increase in *Vglut2* signal strength. **(H–I)** Hemisectioins visualizing BRN3A^+^, marking in the dorsal midbrain postmitotic glutamatergic neurons indicate a significant increase of these cells in *Dusp16*^−/−^ mutants WT (*n* = 4; *Dusp16*^−/−^; *n* = 4). **(J,J′)** H&E coronal hemisections at the level of future sensorimotor cortex of E18.5 WT and *Dusp16*^−/−^ mutants illustrate an increase in cerebral cortex thickness in mutants. **(K)** Higher magnification of the mediolateral cortex marked by the box in **(A)** illustrate cortical thickening in *Dusp16*^−/−^ mutants. **(L)** Quantification of total neocortical area shows significant increases in overall cortical thickness of *Dusp16*^−/−^ mutants. All the values are normalized to 100% which represents the average value for cortex thickness in WT littermate controls (*n* = 3; *Dusp16*^−/−^; *n* = 4). Scale bar: **(C′**,**E)** 200 μm; **(D)** 50 μm; **(H)** 500 μm. C, caudal; CP, cortical plate; IZ, intermediate zone; R, rostral; SVZ, subventricular zone; SP, sublate; VZ, ventricular zone; LV, lateral ventricle; IIIV, third ventricle; Aq, Aqueduct of Sylvius.

During embryogenesis, *Dusp16*^−/−^ mutants exhibited an increase in brain parenchyma (Figures 3A–C′). Quantification of the brain surface area, excluding the ventricles, of *Dusp16*^−/−^ mutants, revealed a significant increase in brain tissue volume (paired sample Student’s *t*-test, *t*_(4)_ = 2.77; *p* = 0.034; Figure 3D). Based on prominent *Dusp16* expression in the dorsal midbrain and the midbrain aqueductal stenosis, we examined this brain region in more detail. The superior colliculus, in the dorsal midbrain, was clearly enlarged in *Dusp16*^−/−^ mutants (Figures 3E,E′). Moreover, the strength of the *in situ* hybridization signal for *Vglut2* and *Gad1*, marking glutamatergic and gabaergic neurons respectively, were increased in the dorsal midbrain, without any apparent changes in the size ratio of both populations (Figures 3F–G′). The quantification of glutamatergic neurons using BRN3A as a marker for postmitotic glutamatergic neurons indicated a 29.13% increase in these cells in *Dusp16*^−/−^ mutants (Student’s *t*-test, *p* = 0.034; Figures 3H–I). The thickness of the posterior parts of the cerebral cortex was reduced in mutants due to the increased pressure caused by the hydrocephalus. In contrast, the rostral parts of the cerebral cortex in the mutant mice showed at the level of the future sensimotor cortex enlarged ventricular, subventricular and intermediate zones (VZ, SVZ, IZ; Figures 3J–K). The total neocortical area was significantly enlarged in the *Dusp16*^−/−^ mutants by 23.36% (Student’s *t*-test, *p* = 0.015; Figure 3L). Taken together, these results show that a mutation in *Dusp16* leads to an increase in brain volume, and support involvement of *Dusp16* in the regulation of brain size.

### The Midbrain Roof Plate and Ependymal Cells Develop Properly in the Absence of *Dusp16*

**Figure 4:**
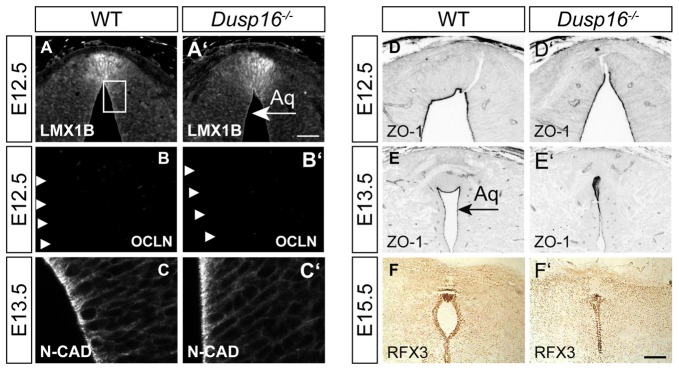
The midbrain roof plate and ependymal cells develop properly in the absence of *Dusp16*. Cell type- and intercellular junction-specific antibodies were visualized by fluorescent or peroxidase-conjugated secondary antibodies in order to study the intactness of the dorsal diencephalon/mesencephalon of WTs **(A–F)** and *Dusp16*^−/−^ **(A′–F′)** at E12.5 and E13.5. **(A,A′)** LMX1B at the roof plate is not altered in mutants. **(B,B′)** The neuroepithelial marker OCLN was not detected in mutants at E12.5, indicating the proper differentiation of radial glia cells from neuroepithelial cells. **(C,C′)** Adherens junctions did not show any differences between genotypes, as assessed by N-CAD expression at the apical surface of radial glia cells at E13.5. **(D–E′)** The adherens junction molecule Zonula occludens-1 (ZO-1), expressed on the apical side of the radial glia cells that face the lumen was present and showed the dorso-ventral expression gradient in mutants as well as in WTS. **(F,F′)** RFX3 required for the differentiation of ependymal cells did not show any aberrations in *Dusp16*^−/−^ mutants. Scale bar: 200 μm. Aq, Aqueduct of Sylvius.

In order to study the mechanisms responsible for the observed closure of the aqueduct, we assessed the integrity of cellular components at the midbrain aqueduct frequently associated with hydrocephalus in other animal models. The roof plate is an important organizer for midbrain development, and perturbations in this structure have been shown to cause hydrocephalus (Fernández-Llebrez et al., 2004). To determine if the roof plate is properly formed in *Dusp16*^−/−^ mutants, we studied the expression of the transcription factor LMX1B. Immunostaining was performed at E12.5, shortly before onset of ventricular enlargement in *Dusp16*^−/−^. Using LMX1B as a roof plate marker, no abnormalities were observed (Figures 4A,A′). Next, we assessed the expression of OCLN, a tight junction marker, exclusively expressed on the apical side of neuroepithelial cells, and not expressed by radial glia cells (Götz and Huttner, 2005). WT and *Dusp16*^−/−^ roof plate cells did not express OCLN at E12.5, indicating that neuroepithelial cells had properly differentiated into radial glial cells (Figures 4B,B′). Radial glial cells form intercellular junctions at the apical side and basal lamina at the basal side which are essential for maintenance of the neural tube morphology. In order to investigate if this inner barrier was properly formed, we assessed the presence of the adherens junction molecule, N-cadherin (N-CAD). Although the dorsal midline exhibited structural differences in *Dusp16*^−/−^ mutants, N-CAD expression was found at the apical surface of radial glia cells of *Dusp16*^−/−^ mutants at E13.5 (Figures 4C,C′). We also assessed the presence of another adherens junction molecule ZO-1, that is expressed on apical side of radial glia cells that face the lumen (Petrov et al., 1994). The lack of Dusp16 in mutants did not affect the presence or dorso-ventral expression gradient of the apical junction molecule ZO-1 (Figures 4D–E′). Finally, we examined RFX3, required for the differentiation of ependymal cells that contribute to CSF homeostasis, and who’s absence causes congenital hydrocephalus in mice (Baas et al., 2006). We observed expression of RFX3 at E15.5 in WT and *Dusp16*^−/−^ mutants and found no difference (Figures 4F,F′). In summary, neither dorsal mis-patterning nor changes in the ependymal development that could have accounted for the aqueductal obstruction were observed in *Dusp16*^−/−^ mutants.

### The Midbrain Sylvian Aqueduct in *Dusp16*^−/−^ Mutants Is Obstructed by Neurons

**Figure 5:**
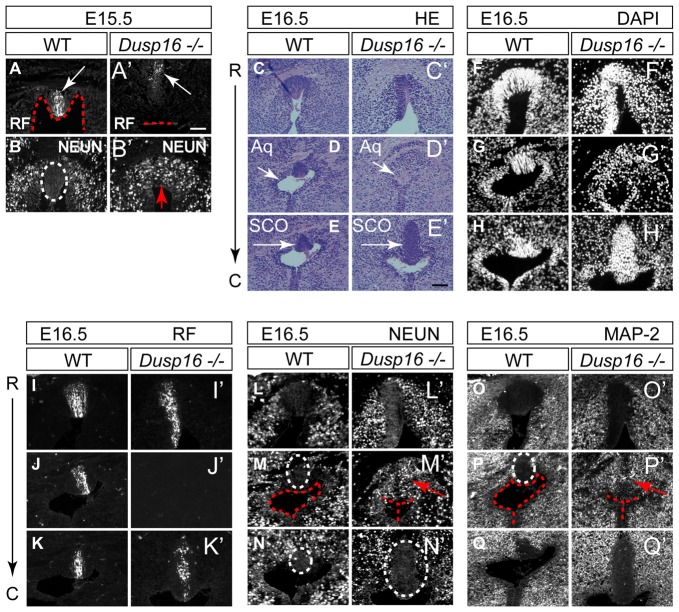
The sylvian aqueduct in *Dusp16*^−/−^ mutants is obstructed by neurons. **(A–B′)** Immunostaining of E15.5 mouse SCO reveals RF immunoreactivity (white arrow) directly at the luminal surface (red dotted line) in WT **(A)** compared with *Dusp16*^−/−^ mutants where RF (white arrow) did not reach the lumen (red dotted line; **A′**). In WTs littermates, the midline SCO (white dotted circle) was devoid of neurons **(B)** compared to *Dusp16*^−/−^ mutants, where NEUN^+^ cells were found in the midline (red arrow; **B′**), in the region that was devoid of RF. **(C–Q′)** Consecutive coronal sections from rostral (R) to caudal (C) levels of WT and *Dusp16*^−/−^ at E16.5. **(C–E′)** H&E staining, **(F–H′)** DAPI staining, **(I–K′)** RF immunoreactivity, **(L–N′)** NEUN immunoreactivity, **(O–Q′)** MAP-2 immunoreactivity, indicate the site of obstruction in *Dusp16*^−/−^ is filled with neurons (red arrow). Red dotted line in **(A,A′,M,M′,F,F′)** indicates ventricular lumen in WTs and closed lumen in mutants. White dotted circle in **(B,M,M′,N,N′,P)** indicate SCO in WTs and mutants. Red arrows in **(B′,M′,P′)** indicate ectopic neurons in the midline of *Dusp16*^−/−^ mutants. Scale bars: **(A′)** 100 μm; **(C–Q′)** 200 μm. Aq, Aqueduct of Sylvius; SCO, subcommisural organ.

The subcommissural organ (SCO), located at the entrance of the midbrain aqueduct, is a brain gland producing RF that is critical to prevent the collapse of the aqueduct (Vio et al., 2000). Although RF was made by *Dusp16*^−/−^ mutants at the site of obstruction, it did not reach the lumen (Figures 5A,A′). Unexpectedly, the obstructed area in mutants, directly ventral to the RF producing cells, was filled with neurons, marked by NEUN (Figures 5B,B′). We then studied consecutive sections of E16.5 WT and *Dusp16*^−/−^ mutants stained with H&E, DAPI visualizing cell nuclei, MAP-2 and NEUN as neuronal markers and RF. Although at the site of obstruction SCO cells seemed to be missing, a significant increase in SCO cells directly posterior to the stenosis indicated that SCO had been relocated (Figures 5C–H′). This finding was further supported by using an RF specific antibody (Rodríguez et al., 1984; Figures 5I–K′), indicating that RF were formed, but posteriorly displaced. The ectopic expression of the neuronal markers, MAP-2 and NEUN to the midline of *Dusp16*^−/−^ mutants (Figures 5L–Q′), which in WT littermates was devoid of neurons, suggests that an increase in neurons has caused the dislocation of SCO cells. In sum, the dorsal midline, normally free of neurons, is filled with neurons, at the site of occlusion in *Dusp16*^−/−^ mutants.

### A Delayed Cell Cycle Exit of Neural Progenitors in *Dusp16*^−/−^ Mutants Is the Cause of the Expanded Neural Progenitor Pool

**Figure 6:**
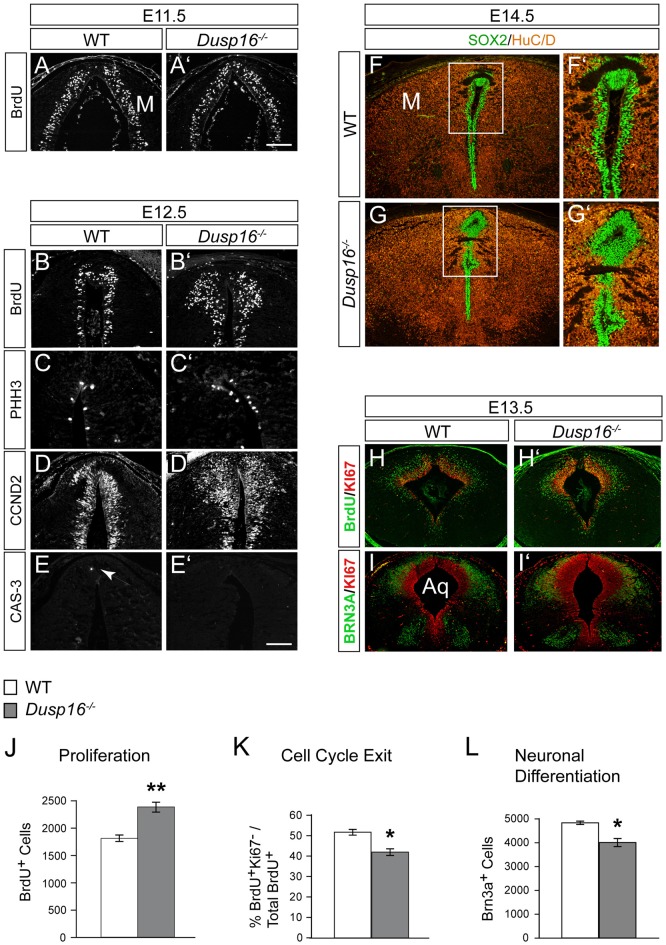
A delayed cell cycle exit of neural progenitors in *Dusp16*^−/−^ mutants is the cause of the expanded neural progenitor pool. **(A,A′)** Immunofluorescence of coronal sections for 5-bromo-2′-deoxyuridine (BrdU) through the dorsal midbrain after 1 h BrdU pulse in WT **(A)** and *Dusp16*^−/−^ **(A′)** E11.5 embryos. **(B–E)** Representative coronal sections through the dorsal midbrain at E12.5 preceding the onset of aqueductal obstruction. **(B,B′)** Immunofluorescent analysis for BrdU after 1 h BrdU pulse in WT **(B)** and *Dusp16*^−/−^ **(B′)** E12.5 embryos showing increase in the number of proliferating radial glia cells in *Dusp16*^−/−^ mutants. **(C,C′)** Immunofluorescent analysis of PHH3^+^ cells reveal increased number of luminal mitotic cells in *Dusp16*^−/−^ mutants compared with WT control littermates. **(D,D′)** Immunofluorescent analysis of CCND2^+^ cells indicates increased number of the cells going through the G_1_ phase of the cell cycle in *Dusp16*^−/−^ mutants. **(E,E′)** No change in apoptosis as measured by phosphorylated CAS-3 immunostaining. Arrow in **(E)** indicates CAS-3^+^ cell in WT. **(F–G′)** At the site of occlusion at E14.5, SOX2^+^ proliferative cells fill the midbrain aqueduct surrounded by HuC/D^+^ early neurons. **(F′,G′)** represent higher magnification of the region depicted by a square in **(F,G)**. **(H–I′)** Double immunofluorescence of the midbrain posterior to the site of obstruction. KI67 (red) and BrdU (green) after 24 h BrdU pulse was used to calculate the fraction of cells exiting the cell cycle at E13.5 in the midbrain. **(I,I′)** Double immunofluorescence for KI67 (green) and BRN3A (red) of WT and *Dusp16*^−/−^ E13.5 embryos. **(J)** Quantification of BrdU^+^ cells throughout entire dorsal midbrain at E12.5 shows significant increase in *Dusp16*^−/−^ mutant mice at E12.5 (WT, *n* = 3; *Dusp16*^−/−^; *n* = 4). Scale bar: 200 μm. **(K)** Quantification of the fraction of cells leaving the mitotic cycle (quitting fraction: percentage of the number of BrdU^+^/KI67^−^ cells among the total of BrdU^+^ cells). In *Dusp16*^−/−^ mutants, the number of cells that leave the cell cycle is significantly reduced compared with control littermates (WT, *n* = 3; *Dusp16*^−/−^; *n* = 4). **(L)** Quantification BRN3A^+^ cells shows significant reduction in *Dusp16*^−/−^ mutant mice at E13.5 in the midbrain (WT, *n* = 3; *Dusp16*^−/−^; *n* = 4). M, midbrain; Aq, Aqueduct of Sylvius.

To better understand the role of *Dusp16* in neurogenesis and how aberrations in this process may cause hydrocephalus, we analyzed proliferation and differentiation of neural progenitors in the dorsal midbrain. Mutants were examined at E11.5 and E12.5, 1 and 2 days before the midbrain aqueduct is obstructed by overproliferating cells (Figures 6A–E′).

To investigate progenitor proliferation, we assessed the number and position of nuclei entering S-phase. Timed-mated pregnant females were given a single intraperitoneal (i.p.) injection of BrdU at E11.5 and E12.5, and sacrificed 1 h later. At E11.5 no changes in cell proliferation were observed in mutant brains compared to control littermates (Figures 6A,A′). However, at E12.5, 1 day before the onset of ventricular enlargements in *Dusp16*^−/−^ mutants, we observed a significant increase in the number of proliferating radial glia cells in *Dusp16*^−/−^ mutants compared to WTs (Figures 6B,B′). The number of BrdU^+^ cells was significantly increased throughout the entire dorsal midbrain in *Dusp16*^−/−^ mutants by 32% (Student’s *t*-test, *p* = 0.0058, *n* = 3 either genotype; Figure 6J). Complementing these findings, there was in *Dusp16*^−/−^ mutants an increase in metaphase cells distributed along the luminal surface as indicated by an increase in the mitotic marker PHH3 (Figures 6C,C′). Moreover, CCND2, which promotes progression through the *G*_1_ phase of the cell cycle, was increased in *Dusp16*^−/−^ mutants at E12.5 (Figures 6D,D′). In contrast, the mutants did not show any changes in the level of the apoptotic marker phosphorylated Caspase-3 (CAS-3) at E12.5 (Figures 6E,E′). Co-labeling for SOX2, a marker for proliferative nuclei and HuC/D as an early neuronal marker at E14.5, demonstrated that the aqueduct was filled and obstructed with SOX2^+^ neural progenitors (Figures 6F–G′).

To more closely examine the role of *Dusp16* in neural differentiation, we monitored cell cycle exit of neural progenitors in the dorsal midbrain. This was done by quantifying the fraction of cells leaving the mitotic cycle. The “quitting fraction” was calculated as the percentage of BrdU^+^/KI67^−^ cells among the total BrdU^+^ cells, subsequent to a single i.p. injection of pregnant dams at E12.5 with BrdU and sacrifice 24 h later. Using this approach, BrdU^+^ cells co-labeled with the mitotic marker, KI67, were identified as still dividing cells. Cells that were BrdU^+^/KI67^−^ where those, that had exited the cell cycle within the previous 24 h (Figures 6H,H′; Lahti et al., 2011). The percentage of cells exiting the cell cycle in *Dusp16*-deficient mice was significantly reduced by 18.76% (Student’s *t*-test, *p* = 0.0149; Figure 6K).

Since this quantitative analysis suggested a shift in progenitor proliferation, we decided to assess the number of differentiated postmitotic glutamatergic BRN3A^+^ in the dorsal midbrain at E12.5 (Figures 6I,I′). Indeed, in contrast to later embryonic stages (Figures 3H,I), the number of BRN3A^+^ cells was significantly reduced by 17.18% in *Dusp16*-deficient mice at midgestation (Student’s *t*-test, *p* = 0.0080; Figures 6I,I′,L). We conclude that lack of *Dusp16* leads to an increase of the neural progenitor pool, by means of decreased cell cycle exit.

### In *Dusp16*^−/−^ Mutants the Numbers of Phospho-p38 Positive Cells Was Increased Together with the Expanded Progenitor Pool

**Figure 7:**
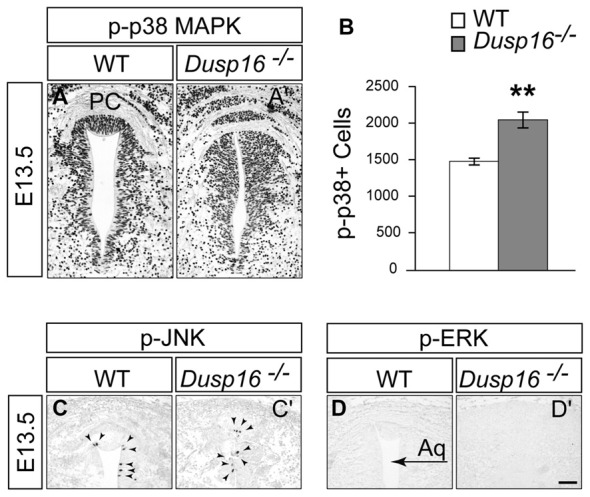
In *Dusp16*^−/−^ mutants the numbers of phospho-p38 positive cells was increased together with the expanded progenitor pool. **(A,A′)** Immunohistochemical staining of coronal sections through the dorsal midbrain of E13.5 embryos visualizing phosphorylated p38 mitogen-activated protein kinase (MAPK), phospho-JNK, and phospho-ERK indicate that there is a strong phosphorylated p38 MAPK staining in the VZ of *Dusp16*^−/−^ mutants compared to control littermates. **(B)** There is a significant increase in phospho-p38 MAPK positive cells in mutants (WT, *n* = 3; *Dusp16*^−/−^; *n* = 3) **(C,C′)**. In contrast, phospho-JNK is only found in a few cells in the ventricular lining (arrowheads), with no changes between the genotypes. **(D,D′)** No signal is detectable for phospho-ERK in WTs as well as in *Dusp16*^−/−^ mutants. Scale bar: 200 μm. PC, posterior commissure.

Since ERK, JNK and p38 MAPK are the major substrates for DUSPs, we assessed their phosphorylation in the dorsal midbrain at E13.5, when the hydrocephalus is first manifest (Bermudez et al., 2010). Strong phosphorylated p38 MAPK staining was observed concentrated in the VZ of mutants (Figures 7A,A′). Comparing WTs with mutants revealed a significant increase in the number of phospho-p38 MAPK positive cells in the VZ of the latter by 37.61% (Student’s *t*-test, *p* = 0.008; Figure 7B). In contrast, only a few phospho-JNK positive cells were observed in this region in both, WTs and *Dusp16*^−/−^ mice, without apparent differences between genotypes despite the strong increase in ventricular cells (Figures 7C,C′). phospho-ERK staining was not detected in the region of interest (Figures 7D,D′). In conclusion, these data suggest that the MAPK most likely involved in the *Dusp16*^−/−^ phenotype is p38 MAPK.

## Discussion

### Overproliferation Causing Congenital Obstructive Hydrocephalus in *Dusp16*^−/−^ Mutants

Hydrocephalus is a complex disorder involving multiple genetic and environmental components. Thus, many pathways and mechanisms are likely to be implicated in its etiology and pathophysiology. Dysfunction of the SCO and abnormalities in ependymal cells has been suggested to be major causes of congenital obstructive hydrocephalus (Huh et al., 2009; Jiménez et al., 2014). Aberrations in neural stem and progenitor cell proliferation and survival have also been associated with hydrocephalus. Thus, several reports have demonstrated a reduction of neural progenitor proliferation as well as increased cell death in hydrocephalus. As underlying associated mechanisms abnormal cell-cell junctions between neural stem cells and ependymal cells has been variously reported to induce loss of these cells and their and detachment from the VZ (Domínguez-Pinos et al., 2005; Rodríguez et al., 2012). Subsequently, the disruption of the VZ in the walls of the Sylvian aqueduct leads to obliteration and stenosis during embryonic development (McAllister, 2012). Impaired neural progenitor proliferation and loss of neural stem cells have also been associated with non-obstructive hydrocephalus in a mouse ciliopathy model as well as with other genetic hydrocephalus models (Tian et al., 2011; Carter et al., 2012).

Lack of *Dusp16* expression in the CP and the normal morphology of this structure, suggest that CSF overproduction is not the cause of hydrocephalus in *Dusp16*^−/−^ mutants. Moreover, the lack of dilatation of the fourth ventricle suggests that impaired reabsorption of CSF into the venous blood stream is also not responsible for the ventricular dilatations in these animals. In contrast, the occlusion of the midbrain aqueduct, preceding ventricular enlargement, strongly suggests that this is the cause of the hydrocephalus in *Dusp16*^−/−^ mutants.

In *Dusp16*^−/−^ mutants we did not find any abnormalities in ependymal cells and cell junctions. Similarly, the SCO was formed and produced RF, leading us to conclude that the overproliferation of neural progenitor cells we observed causes the hydrocephalus in *Dusp16*^−/−^ mutants. This is strengthened by our finding that progenitor cells and, later during development, mature neurons, obstruct the midbrain aqueduct. Importantly, we observed that proliferation of neural progenitor cells is first seen shortly before the onset of occlusion. A reprogramming of SCO cells to neurons seems unlikely as RF producing SCO cells are not reduced, but are, rather, moved posterior to the occlusion by over-proliferating cells. Thus our data support obstruction of the midbrain aqueduct by over-proliferation of neural progenitors as the proximal cause of hydrocephalus in *Dusp16*^−/−^ mutants.

### A Potential Role for *Dusp16* in the Combination of Hydrocephalus and Brain Overgrowth Disorders

The particular combination of hydrocephalus, brain overgrowth and abnormal MAPKs signaling, found in *Dusp16*^−/−^ mutants, recapitulates aspects of different neurodevelopmental disorders. Megalencephaly-polymicrogyria-polydactyl-hydrocephalus (MPPH) syndrome in humans is a predominantly prenatal developmental syndrome characterized by megalencephaly and bilateral perisylvian polymicrogyria, caused by overproliferation of the dorsal diencephalon and midbrain. Hydrocephalus and polydactyly are more variable features seen in nearly half of the reported individuals. *De novo* germline mutations in three core PI3K-AKT-mTOR pathway genes are now known to be associated with MPPH including, PIK3R2, AKT3 and CCND2 (Mirzaa et al., 2014). CCND2 plays a major role in neural progenitor proliferation and its increased expression in *Dusp16*^−/−^ embryos indicates that it is part of the molecular mechanism causing neural overproliferation in these mutants. The phosphorylation and subsequent inactivation of CCND2 by GSK3-beta plays a key role in controlling CCND2 activity, and dysregulation of this interaction has been implicated in megalencephaly (Huang et al., 2007; Kida et al., 2007; Mirzaa et al., 2014). Inhibition of GSK3-beta activity is the principle mechanism regulating this kinase. Besides inhibition by the protein kinase Akt at the N terminus (Ser^9^), p38 MAPK also inactivates GSK3-beta by direct phosphorylation at its C terminus (Thr^389^), occurring primarily in the brain and in thymocytes (Thornton et al., 2008). These findings suggest the possibility that in *Dusp16*^−/−^ mutants altered p38 MAPK / GSK3-beta interaction leads to an increased CCND2, which has previously been causally linked to MPPH.

The combination of MAPK pathway abnormalities, hydrocephalus and brain overgrowth is also observed in RASopathies. This clinically-defined group of syndromes is caused by germline mutations in genes encoding components or regulators of the Ras/MAPK pathway. Many of these disorders show brain overgrowth combined with hydrocephalus. They include Noonan syndrome, Costello syndrome, cardiofaciocutaneous syndrome and neurofibromatosis, type I (Winden et al., 2015; Kousi and Katsanis, 2016). For the phenotype analysis of *Dusp16*^−/−^ mutants reported here, we focused on changes in embryonic CNS development. A future, detailed analysis of the phenotype of these mutants outside of the CNS will provide necessary information, to follow up a potential role of DUSP16 in RASopathies. Taken together, DUSP16 is a promising candidate gene for a broad spectrum of disorders combining hydrocephalus, brain overgrowth and abnormal MAPK signaling.

### Factors Upstream and Downstream of DUSP16 in Neurogenesis

Along with others, we have demonstrated that growth factors, including Bmps, Fgfs and Wnts, are the essential extracellular regulators of neurogenesis in the midbrain by balancing neural progenitor proliferation and differentiation (Panhuysen et al., 2004; Saarimäki-Vire et al., 2007; Paridaen and Huttner, 2014). A series of evidence indicates that growth factors induce the expression of different *Dusps* including *Dusp3* and *Dusp6* during embryogenesis. In these experiments, the upregulation of *Dusps* serves as a negative feedback regulator of growth factor signaling (Li et al., 2007; Finelli et al., 2013). As would have been expected from this antagonism, the conditional inactivation of Fgf receptors 1 and 2 leads to a phenotype complementary to the one observed in *Dusp16*^−/−^ mutants. While in Fgf receptor mutants there is premature neuronal differentiation and depletion of the progenitor pool (Lahti et al., 2011), *Dusp16*^−/−^ mutants show an increase in the progenitor pool together with delayed neural differentiation. Due to the regulatory role of the MAPK pathway that integrates different signaling pathways, it seems likely that DUSPs are not only involved in modulating Fgf signaling in the developing nervous system, but also serve to integrate signals from different growth factors.

Depending on the cellular context and type of growth factor involved, *Dusp16* appears to differentially impact on cellular proliferation, since bone marrow cell proliferation in response to GM-CSF was reduced in *Dusp16*^−/−^ mice (Niedzielska et al., 2014) and shRNA knockdown in several cancer cell lines induced cellular senescence and cell cycle arrest (Zhang et al., 2015). The mechanistic basis underlying these strikingly different outcomes of *Dusp16*-deficiency remains to be determined, but may be related to the specific MAPK proteins involved.

Classical DUSPs containing a MAPK-binding domain have high substrate specificity for MAPKs, with *Dusp16* showing a preference for JNK and p38 MAPK (Masuda et al., 2001; Matsuguchi et al., 2001; Tanoue et al., 2001; Theodosiou and Ashworth, 2002). Based on the limitations of immunohistochemistry for the quantification of signaling components, we cannot prove by which substrate *Dusp16* regulates neurogenesis. However, the abundant phosphorylation of p38 MAPK in *DUSP16*^−/−^ mutants and unaltered levels of phosphor-JNK suggest that p38 MAPK is an obvious candidate mediating the effects of *Dusp16* on neurogenesis. p38 MAPK is activated by various cytotoxic stresses, cytokines and growth factors (Cuadrado and Nebreda, 2010; Coffey, 2014). In contrast to JNK signaling, which has been shown to negatively regulate neuronal progenitor proliferation (Coffey, 2014), the role of p38 MAPKs in neurogenesis is less well understood. In particular, the role of p38 MAPK in neural stem cell proliferation remains controversial. p38 MAPK was initially shown to increase proliferation in an immortalized neuronal cell line (Kim et al., 2008) as well as in adult hippocampal stem cells (Zhang et al., 2011). In contrast, p38 MAPK was also shown to decrease proliferation in neural progenitors in primary embryonic neuronal cultures (Sato et al., 2008) and in neurosphere cultures of adult hippocampal cells (Yoshioka et al., 2015). Based on our study p38 is a promising candidate to mediate the effects of *Dusp16* on neural development. Future studies will provide necessary information about the functional role of Dusp16 regulated p38 activity in neural development and disorders.

In conclusion, we provide evidence that *Dusp16* is necessary for proper neural progenitor cell pool expansion, by regulating their cell cycle exit. We further demonstrate that *Dusp16* deficiency causes and combines congenital obstructive hydrocephalus and brain overgrowth. Moreover, our results establish *Dusp16*^−/−^ mutants as a powerful tool to study neurobiology and novel treatment strategies and suggest that *Dusp16* is a promising candidate gene for human genetic studies investigating these pathologies.

## Author Contributions

KZ, JP, RHF, RL and CB: conceived and designed the experiments. KZ, VMJ, ZV, MN, LK and MMJ: performed the experiments. KZ, VMJ, MN, LK, MMJ, JP, RHF, RL and CB: analyzed the data. KZ and CB: wrote the article.

## Conflict of Interest Statement

The authors declare that the research was conducted in the absence of any commercial or financial relationships that could be construed as a potential conflict of interest.

## Footnotes

^1^http://www.genepaint.org
