# Corrigendum: Dusp16 Deficiency Causes Congenital Obstructive Hydrocephalus and Brain Overgrowth by Expansion of the Neural Progenitor Pool

**DOI:** 10.3389/fnmol.2018.00029

**Published:** 2018-02-01

**Authors:** Ksenija Zega, Vukasin M. Jovanovic, Zagorka Vitic, Magdalena Niedzielska, Laura Knaapi, Marin M. Jukic, Juha Partanen, Roland H. Friedel, Roland Lang, Claude Brodski

**Affiliations:** ^1^Department of Physiology and Cell Biology, Zlotowski Center for Neuroscience, Faculty of Health Sciences, Ben-Gurion University of the Negev, Beersheba, Israel; ^2^Institute of Clinical Microbiology, Immunology and Hygiene, University Hospital Erlangen, Friedrich-Alexander-Universität Erlangen-Nürnberg, Erlangen, Germany; ^3^Department of Biosciences, University of Helsinki, Helsinki, Finland; ^4^Departments of Neuroscience and Neurosurgery, Friedman Brain Institute, Icahn School of Medicine at Mount Sinai, New York, NY, United States

**Keywords:** DUSP16, hydrocephalus, brain overgrowth, megalencephaly, macrocephaly, neurogenesis, neural differentiation, neuronal progenitors

An author name was incorrectly spelled as Roland F. Friedel. The correct spelling is Roland H. Friedel. The authors apologize for this error and state that this does not change the scientific conclusions of the article in any way.

The original article has been updated.

## Conflict of interest statement

The authors declare that the research was conducted in the absence of any commercial or financial relationships that could be construed as a potential conflict of interest.

